# Traumatic Axonal Injury in the Optic Nerve: The Selective Role of SARM1 in the Evolution of Distal Axonopathy

**DOI:** 10.1089/neu.2022.0416

**Published:** 2023-08-16

**Authors:** Athanasios S. Alexandris, Youngrim Lee, Mohamed Lehar, Zahra Alam, James McKenney, Dianela Perdomo, Jiwon Ryu, Derek Welsbie, Donald J. Zack, Vassilis E. Koliatsos

**Affiliations:** ^1^ Department of Pathology, Johns Hopkins University School of Medicine, Baltimore, Maryland, USA.; ^2^ Department of Otolaryngology—Head and Neck Surgery, Johns Hopkins University School of Medicine, Baltimore, Maryland, USA.; ^3^ Viterbi Family Department of Ophthalmology and Shiley Eye Institute, University of California San Diego, La Jolla, California, USA.; ^4^ Department of Ophthalmology, Johns Hopkins University School of Medicine, Baltimore, Maryland, USA.; ^5^ Department of Molecular Biology and Genetics, Johns Hopkins University School of Medicine, Baltimore, Maryland, USA.; ^6^ Department of Neuroscience Johns Hopkins University School of Medicine, Baltimore, Maryland, USA.; ^7^ Department of Genetic Medicine, Johns Hopkins University School of Medicine, Baltimore, Maryland, USA.; ^8^ Department of Neurology, Johns Hopkins University School of Medicine, Baltimore, Maryland, USA.; ^9^ Department of Psychiatry and Behavioral Sciences, Johns Hopkins University School of Medicine, Baltimore, Maryland, USA.

**Keywords:** neurodegeneration, neuroinflammation, stereology, traumatic brain injury, Wallerian degeneration

## Abstract

Traumatic axonal injury (TAI), thought to be caused by rotational acceleration of the head, is a prevalent neuropathology in traumatic brain injury (TBI). TAI in the optic nerve is a common finding in multiple blunt-force TBI models and hence a great model to study mechanisms and treatments for TAI, especially in view of the compartmentalized anatomy of the visual system. We have previously shown that the somata and the proximal, but not distal, axons of retinal ganglion cells (RGC) respond to DLK/LZK blockade after impact acceleration of the head (IA-TBI). Here, we explored the role of the sterile alpha and TIR-motif containing 1 (SARM1), the key driver of Wallerian degeneration (WD), in the progressive breakdown of distal and proximal segments of the optic nerve following IA-TBI with high-resolution morphological and classical neuropathological approaches. Wild type and *Sarm1* knockout (KO) mice received IA-TBI or sham injury and were allowed to survive for 3, 7, 14, and 21 days. Ultrastructural and microscopic analyses revealed that TAI in the optic nerve is characterized by variable involvement of individual axons, ranging from apparent early disconnection of a subpopulation of axons to a range of ongoing axonal and myelin perturbations. Traumatic axonal injury resulted in the degeneration of a population of axons distal and proximal to the injury, along with retrograde death of a subpopulation of RGCs. Quantitative analyses on proximal and distal axons and RGC somata revealed that different neuronal domains exhibit differential vulnerability, with distal axon segments showing more severe degeneration compared with proximal segments and RGC somata. Importantly, we found that *Sarm1* KO had a profound effect in the distal optic nerve by suppressing axonal degeneration by up to 50% in the first 2 weeks after IA-TBI, with a continued but lower effect at 3 weeks, while also suppressing microglial activation. *Sarm1* KO had no evident effect on the initial traumatic disconnection and did not ameliorate the proximal optic axonopathy or the subsequent attrition of RGCs, indicating that the fate of different axonal segments in the course of TAI may depend on distinct molecular programs within axons.

## Introduction

Traumatic brain injury (TBI), a common problem with close to 3 million casualties requiring medical attention annually, is not only a major clinical concern in and of itself, but may also form the biological nidus for neurodegenerative disease and serve as a clinical model for these conditions.^1^ A very common pathology in TBI of various causes and degrees of severity is diffuse or traumatic axonal injury (DAI or TAI) that is thought to be caused by rotational acceleration of the head and extremely rapid axonal stretching in multiple white matter tracts in the forebrain and brain stem.^2–7^ Although classically encountered in the human and other gyrencephalic brains featured by a disproportionate abundance of white matter tracts,^3,4,6-8^ we and others have shown that TAI can be modeled in the rodent brain with specially delivered impact forces.^5,9–14^

TAI in the optic nerve (ON) and optic tract is a consistent finding in multiple blunt-force TBI models such as fluid percussion and impact acceleration (IA) and also in models of blast, suggesting that the visual system is exceedingly vulnerable in multiple injury situations.^5,15–20^ This interesting observation, combined with the accessibility and compartmentalized anatomy of the visual system, suggests that retinal ganglion cells (RGCs) and their axons and terminals are an excellent neuronal system in which to model TAI and explore the underlying cellular and molecular mechanisms of injury.

Our prior research on the visual system after diffuse TBI has disclosed similarities between TAI and simpler forms of axonal injury such as axotomy. This includes the dependency of retrograde degeneration of RGCs and in part, axonopathy, on signaling by members of the stress-activated mitogen activated protein kinase (MAPK) cascade, specifically the MAP3Ks DLK (dual leucine zipper kinase, or MAP3K12) and LZK (leucine zipper kinase, or MAP3K13).^20-22^ The proximal, but not distal traumatic axonopathy appears to respond to DLK/LZK blockade.^22^

Wallerian degeneration (WD) is a highly conserved process of axonal self-destruction triggered by the activation of the NAD+ hydrolase sterile alpha and TIR motif containing 1 (SARM1).^23^ Wallerian degeneration is initiated with the arrest in transport and degradation of the labile NAD^+^-synthesizing enzyme nicotinamide nucleotide adenylyltransferase 2 (NMNAT2) by members of the stress MAPK cascade and the atypical ubiquitin E3 ligase complex MYCBP2 -SKP1-FBXO45.^24,25^ Loss of NMNAT2 and the resulting disequilibrium between NAD^+^ and its precursor NMN appear to be required for the activation of SARM1 and the subsequent breakdown of the axon.^25-27^

It has been recently shown that *Sarm1* deletion can reduce the pathological burden associated with traumatic axonopathy,^12,28,29^ but this work was limited by the inclusion of mixed populations of axons with lack of clear demarcation between proximal and distal axons that may engender different cellular and molecular responses to injury. Here, we explore the role of SARM1 in both the distal and proximal segments of optic nerves injured by impact acceleration TBI (IA-TBI) at acute and chronic time-points after injury using high-resolution morphological and classical neuropathological approaches. The separation between axon segments proximal and distal to the traumatic biomechanical disruption^16,20,22^ and the use of a time course of changes allows for a better appreciation of the dynamic role of WD signaling in different domains of injured axons.

## Methods

### Experimental subjects and impact acceleration TBI model

Mice were 10 to 14-week-old male C57BL/6 mice, transgenic *Sarm1* KO mice backcrossed to the C57BL/6 background^30^ (RRID:MGI:5507810; gift from Dr. A. Hoke, Johns Hopkins School of Medicine), B6.Cg-Tg(Thy1-YFP)HJrs/J (referred here as YFP-H; RRID:IMSR_JAX:003782) and YFP-H/*Sarm1* KO double transgenic mice. *Sarm1* KO mice were produced with homozygous breeding and congenic C57BL/6 mice were used as controls (referred here as wild type [wt]). It should be noted that while *Sarm1* KO mice have been backcrossed to the C57BL/6 strain, recent analysis revealed that they may have genetic variations in neighboring genes belonging to the original 129 strain.^31^ However, the specific effect of *Sarm1* KO on axon degeneration *in vivo* has been confirmed by CRISPR/Cas9 editing^31^ and the expression of dominant negative constructs.^32^ Mice were subjected to IA-TBI or sham injury (Table 1) as described with slight modifications.^12,22,33^ Briefly, mice were anesthetized with a mixture of isoflurane, oxygen and nitrous oxide, the cranium was exposed, and a 5 mm-thick stainless-steel disc was glued onto the skull midway between bregma and lambda sutures. Then, a 50 g weight was dropped from 85 cm on the metal disk, while the mouse was placed on a foam mattress (4–0 spring constant foam; Foam to Size Inc., Ashland, VA), with the body immobilized with tape. Immediately after injury, the disc was removed and the skull was examined for skull fractures; the rare animals with fractures (< 2%) were excluded. The scalp incision was closed with surgical staples. Sham animals underwent the same procedure without the weight drop component. Neurological recovery was assessed by the presence and duration of apnea or irregular breathing and the revival of the righting reflex. Subjects with apnea/irregular breathing >150 sec and time-to-righting reflex <50 sec or >550 sec were excluded (< 5%). After recovery, animals were returned to their vivarium with a 12-h light/12-h dark cycle and *ad libitum* access to food and water. Surgical procedures and injuries were performed under aseptic conditions and all animal handling and postoperative procedures were carried according to protocols approved by the Animal Care and Use Committee of the Johns Hopkins Medical Institutions (Protocol Number: MO19M458).

**Table 1.** Summary of Experimental Groups and Histological Procedures

Experiment	Mouse cohorts	Methods
Blood–brain barrier assessment	YFP-H (*n* = 6); survival 15 min or 4 h post IA-TBI or sham injury.	Tissue clearing and confocal imaging
Assessment of acute disconnection	YFP-H and YFP-H/*Sarm1* KO (*n* = 10); survival 24 h post IA-TBI.	Tissue clearing and confocal imaging
Ultrastructural assessment of traumatic	C57BL/6 (*n* = 6); survival 15 min or 4 h.	Electron microscopy
axonal injury Ultrastructural and stereological assessment of traumatic axonopathy	C57BL/6 ; *Sarm1* KO (*n* = 92); survival 3, 7, 14, and 21 days post IA-TBI or sham injury. Additional wt and SKO mice at 3, 7, and 21 days were also prepared for further ultrastructural analyses (*n* = 13).	Electron microscopy, stereology on toluidine blue stained semithin sections.
Assessment of neuroinflammation	C57BL/6, *Sarm1* KO (*n* = 33); survival 7 and 28 days post IA-TBI or sham injury.	Tissue clearing and confocal imaging

IA-TBI, impact acceleration traumatic brain injury; wt, wild type; SKO, *Sarm1 knockout.*

### Blood–brain barrier assessment

For the blood–brain barrier (BBB) disruption studies, YFP-H mice (*n* = 2 per time-point) received IA-TBI or sham injury and were injected intraperitoneally with 400 μL of 20 mg/mL EZ-Link™ Sulfo-NHS-LC-Biotin (Thermo Fisher Scientific, Cat. #21335), 20 min before perfusion with 4% paraformaldehyde (PFA) in phosphate-buffered saline (PBS). Formaldehyde fixed optic nerves were processed with the SHIELD protocol^34^ for tissue clearing as per manufacture instructions (LifeCanvas Technologies, MA; Cat.# C-PCK-250-1.52). For the visualization of EZ-Biotin, ONs were incubated with Streptavidin, Alexa Fluor™ 594 conjugate (1:1000; Thermo Fisher Scientific, Cat. #S32356), before washing and index matching. Optic nerves were then imaged with a ZEISS 880 Airyscan microscope using 10 × , 25 × and 40 × objectives.

### Tissue clearing for optic nerve anatomy

To assess the three-dimensional anatomy of ON axons and its alteration shortly after injury in the presence or absence of *Sarm1*, we crossed YFP-H mice with *Sarm1* KO mice and subjected them to IA-TBI. After perfusion fixation, one of the two ONs was selected at random and processed for clearing and for imaging at 20 × with a ZEISS 880 Airyscan microscope as described above (*n* = 5 per genotype). An investigator blinded to group designation counted axons manually. The total number of axons per ON was estimated by counting manually the number of axons in the proximal ON segment (at three levels) and then the fraction of non-disconnected axons was estimated from the number of axons passing through the injury front with no interruption in YFP-H signal.

### Tissue preparation for semithin section analysis and transmission electron microscopy

Wild-type C57BL/6 and *Sarm1* KO mice were randomly allocated to sham or IA-TBI condition, and to different survival end-points (3, 7, 14, and 21 days; *n* = 8-10 per time-point, per genotype). Mice were transcardially perfused with 2% paraformaldehyde and 2% glutaraldehyde in 50 mM sodium cacodylate, 50 mM phosphate and 3 mM magnesium chloride buffer (pH = 7.4) for 30 min. Tissues were left in situ at room temperature for 2 h before dissection and incubated overnight at 4°C in the same fixative. Tissues were rinsed (5 × 15 min) in 75 mM sodium cacodylate, 75 mM phosphate, and 3 mM magnesium chloride (pH = 7.4) on a rotator. To adjust osmolarity, the first two rinsing steps included 2:1 and 1:1 mixture of fixative solution and buffer at 4°C, respectively. Tissues were then incubated in freshly prepared 2% osmium tetroxide with 1.6% potassium ferrocyanide in the same buffer for 2 h at room temperature in the dark.

Following osmication, tissues were rinsed three times in 100 mM maleate, 3.5% sucrose buffer (pH = 6.2) for 10 min and incubated with 2% uranyl acetate in maleate-sucrose buffer for 1 h followed by step-wise rinsing steps in maleate buffer, 1:1 maleate buffer with distilled water, and finally distilled water for 5 min each. Tissue blocks were dehydrated in a graded ethanol series, transferred in propylene oxide and incubated in 50% embedded in EMbed 812 resin (EMS14120, Electron Microscope Sciences, Hatfield, PA). Semithin (1 μm) and thin (60 nm) sections were sectioned in a Leica UC7 ultramicrotome (Leica Microsystems, Deerfield, IL). Sections were obtained through the transverse and longitudinal planes to sample the injury front as well as segments distal and proximal to it. “Distal” sections were obtained at a pre-chiasmatic ON level where all myelinated fibers are visible at cross-section and “proximal” sections immediately distal to the exit of the ON from the optic foramen. Transverse semithin sections were stained with 1% toluidine blue. Thin sections were mounted on square mess copper grids (EMS300-Cu, Electron Microscope Sciences, Hatfield, PA), stained with uranyl acetate and lead citrate, and examined and photographed on a Hitachi H7600 transmission electron microscope (Hitachi High-Technologies Corporation, Tokyo, Japan).

### Stereological quantitation of intact and abnormal myelinated axons

Transverse semithin sections distal and proximal to the site of traumatic biomechanical disruption were stained with toluidine blue. Sections were prepared from one ON per subject that was selected randomly and stereological analysis was performed by two separate investigators blinded to experimental history. Interrater reliability was very high with Pearson's r of 0.997. Analysis was performed at 100 × using systematic random sampling and the optical fractionator probe, with the aid of a motorized stage Axioplan microscope (Carl Zeiss Inc.) and Stereo Investigator® software (Microbrightfield Inc., Williston, VT). Parameters were selected based on empirically determined sampling methods^35^ and were as follows: counting frame size of 4 × 4 μm, grid size of 17.89 × 17.89 μm and sampling fraction of 4%. Pathological profiles included fibers with condensed (dark) or hydropic axoplasm and pathological sheaths with myelin thinning, excess myelin figures, and collapsed myelin.

### Processing of retinas and quantitation of retinal ganglion cell bodies

Because glutaraldehyde fixation poses challenges in immunohistochemistry for RGC markers, we estimated RGC densities using hematoxylin-stained sections. Briefly, eyes were embedded in paraffin after removal of the cornea and sectioned at 10 μm. Sections through the optic nerve head were stained with hematoxylin, mounted with DPX and examined for the identification of RGCs in the ganglion cell layer based on nuclear histology.^36,37^ Retinal ganglion cells are characterized by a large, round, lightly stained nucleus and prominent central nucleolus. Cells with elongated nuclei were deemed to be epithelial cells, while cells with small, dense, and uniformly stained round nuclei were identified as amacrine cells.^36,37^

Cell counts were performed by an investigator blinded to experimental history. Using the optical fractionator stereological probe, the retinal ganglion cell layer was traced at 10 × and individual RGC profiles were counted under 40 × magnification. For each mouse, RGC count was determined as the mean count of three sections.

### Assessment of neuroinflammation in cleared optic nerves

To assess activation of microglia and phagocytosis in ONs after IA-TBI, wild-type, and *Sarm1* KO mice were randomly allocated to sham or IA-TBI condition, and left to survive for 7 or 28 days (*n* = 5-6 per time-point × per genotype). Mice were transcardially perfused with 4% PFA in PBS as in a previous section. Optic nerves were processed with the SHIELD protocol as above with the following change: before, refractive index matching, optic nerves were incubated with a monoclonal rabbit antibody (E4O4W) against IBA1 (1:50; Cell Signaling Technology, Cat# 17198; RRID:AB_2820254) and a rat monoclonal antibody (clone FA-11) against CD 68 (1:100; Bio-Rad Cat# MCA1957GA, RRID:AB_324217) for 4 days at 37°C in PBS with 0.1% Triton (PBST) and 0.1% NaN_3._ Then they were washed three times in PBST over 8 h and further incubated with secondary goat anti-rat Alexa Fluor Plus 647 (Thermo Fisher Scientific Cat# A48265, RRID:AB_2895299) and anti-rabbit Alexa Fluor Plus 594 (Thermo Fisher Scientific Cat# A48284, RRID:AB_2896348) for 2.5 days at 37°C, then washed again in PBST and PBS. Z-stack images (60 μm) through the center of the most distal 1-mm segment were captured at 20 × with a ZEISS 880 Airyscan microscope. The signal of the CD 68 channel was quantified in FIJI (NIH, RRID:SCR_002285) as areal density: z-stack images were converted to binary masks with adaptive thresholding after background subtraction, Gaussian blur and sum Z-projection, and the fraction of signal coverage (areal density) was measured.

### Statistical analysis

Graphpad Prism 9.1 (GraphPad Software, La Jolla, Ca, USA; RRID:SCR_002798) was used for statistical analysis and plotting of figures. Two-way analysis of variance (ANOVA) or mixed effect models (*F_df,df_,* where *df* is degrees of freedom) were used for the assessment of genotype, time and/or location effects with adjustment for multiple independent or pairwise comparisons (*t_df_*) as indicated. For the statistical analysis of CD 68 signal, two-way ANOVA was performed after log-transformation of the data to account for heteroskedasticity. Significance threshold (*p*) was set at 0.05.

## Results

### Impact acceleration-TBI results in primary TAI of the optic nerve

**FIG. 1.**
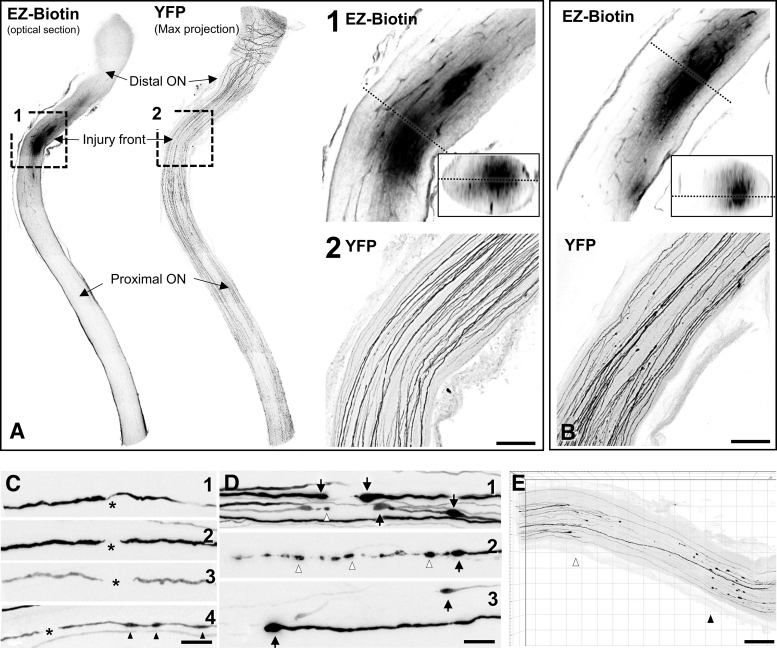
Impact acceleration traumatic brain injury (TBI) results in primary traumatic axonal injury of the distal optic nerve. The acute biomechanical injury is revealed in whole tissue-cleared preparations by extravasation of EZ-Biotin, and morphological changes in YFP-labelled axons. **(A)** Optic nerve (ON) 20 min post-injury. Extravasation of EZ-Biotin indicates a disruption in the blood–brain barrier (BBB) 0.5-1.5 mm proximal to the chiasm, thus dividing the ON in the injury front and the distal and proximal ON segments. Boxes 1 and 2 represent magnifications of rectangular selections on the left shown at 20 × magnification. Small inset in (1) shows orthogonal view through the area marked by dotted line. EZ-Biotin extravasation (1) indicates a greater involvement of the ON core compared with the periphery. The majority of YFP (+) axons passing through the injury front show generally normal morphology (2). **(B)** At 4 h post-injury, the disruption of the BBB (top) is no different in extent than in 20 min (inset shows orthogonal view through the area marked by dotted line), while morphological changes in YFP(+) axons (bottom) are more pronounced. Several axons show extensive discontinuities in YFP signal in an area corresponding to BBB disruption, and the formation of swellings at the proximal and distal borders. (**C, D**) Morphological changes in injured YFP (+) axons at 20 min (C) and 4 h post-injury (D). At 20 min, axons with YFP signal discontinuities (*) may also have swellings or undulations (C_4_, arrowheads). Discontinuities range from 10 to 20 μm and terminal segments tapper off without forming bulbs. At 4 h, morphological changes are more variable; disconnected axons develop proximal and distal terminal bulbs (D_1-3_, arrows). Other axons begin to fragment (white arrowheads in D_1_ and D_2_). Disconnections become more evident than in C, sometimes spanning hundreds of micrometers (D_3_) giving the appearance of axon retraction. **(E)** Imaris-assisted reconstruction of ON 24 h after IA-TBI shows apparent disconnection with retraction of YFP-labeled axons in the proximal (white arrowhead) and distal (black arrowhead) segments. Scale bars: (A, B), 100 μm; (C, D), 20 μm; E, 200 μm.

We have previously shown that IA-TBI results in traumatic injury of RGC axons between the orbital apex and the chiasm as revealed by arrest of cholera toxin B transport, immunoglobulin G extravasation, and the presence of APP (+) undulations, swellings and bulbs.^20,22^ To delineate more accurately the biomechanical insult of IA-TBI on the ON, we visualized the traumatic BBB disruption by a recently developed sensitive technique (i.e., the extravasation of EZ-Biotin)^38^ in combined preparations with ON axonal labeling using Thy1-YFP-H mice (Fig. 1). In these mice, YFP is expressed sparsely in a small population of RGCs, allowing for ON imaging at single-axon resolution. In the first 20 min of IA-TBI, there is focal disruption of the BBB corresponding to an ON segment 0.5-1.5 mm proximal to the chiasm (denoted as “injury front” for the remainder of the article; Fig. 1A). Based on EZ-Biotin signal, BBB disruption is maximal at the ON core, with relative sparing of the periphery (Fig. 1A, 1B).

With respect to pathology in YFP (+) axons, there are minimal changes at 20 min post-injury comprised of occasional discontinuities in YFP signal or undulations (Fig. 1A, 1C) at an area overlapping with the increased EZ-biotin labeling. At 4 h, there is some loss of axonal labeling at or about the injury front and the appearance of swellings proximal and distal to the labeling gap, a pattern suggestive of disconnection with terminal bulbs on either side (Fig. 1B). Compared with the initial disruption at 20 min post-injury, there is further widening of the gap between the apparently disconnected axons, giving the appearance of distal and proximal retraction away from the injury front (Fig. 1B, 1D). The formation of distal and proximal retraction zones bordering the injury front becomes clearer by 24 h (Fig. 1E).

### Ultrastructural features of the evolving traumatic axonopathy in the optic nerve

**FIG. 2.**
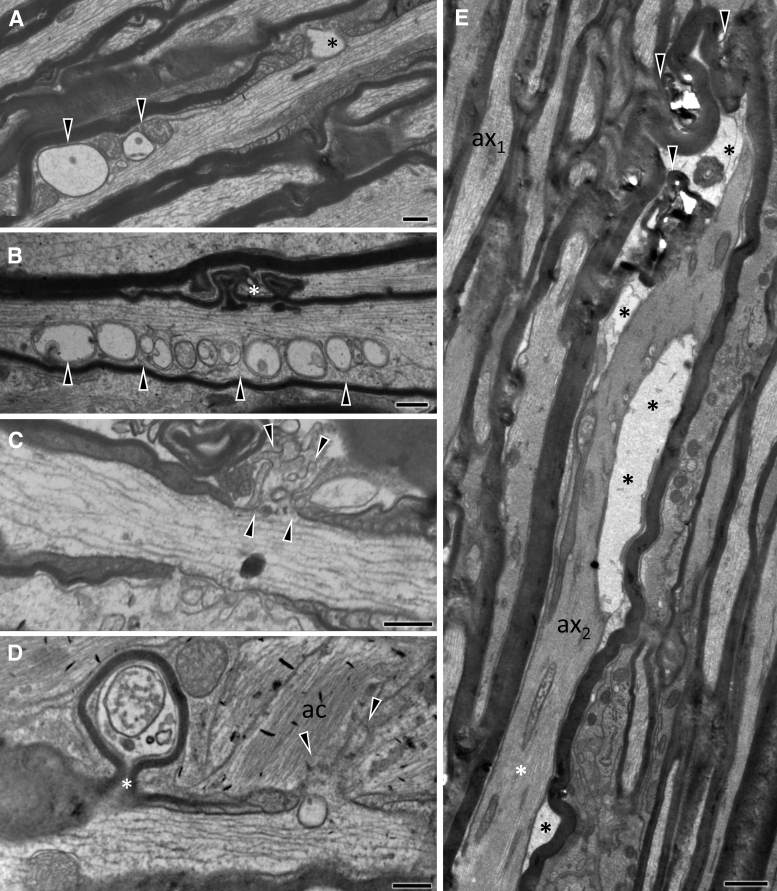
Ultrastructural features of primary traumatic axonal injury of the optic nerve 20 min after impact acceleration traumatic brain injury (IA-TBI). Electron micrographs are taken across the longitudinal plane through the injury front and show a range of axoplasmic, axolemmal and myelin sheath abnormalities. **(A)** Juxtaparanodal accumulation of membrane-bound vacuoles and abnormal mitochondria (arrowheads). A vacuole is also present in the node of Ranvier (*). **(B)** Similar to (A), accumulation of vacuoles and distended mitochondria at an internode (arrowheads). Myelin sheath architecture is disrupted (*). **(C, D)** Nodal blebbing. The axolemma at the node of Ranvier is disrupted leading to extrusion of axoplasm, mostly bound by axolemma (black arrows in both panels). In (D), there is also a large juxta-paranodal protrusion of ensheathed segment of axon (*). **(E)** Severely impacted axonal profile (ax_2_) among normal-appearing axons (ax_1_). The axoplasm in ax_2_ is featured by the formation of hydropic spaces (black asterisks) that contain floccular material and membranes and the compression or compaction of the axoskeleton (white asterisk). The myelin sheath is also disrupted (arrowheads). Ac, astrocyte. Scale bars: (A-D), 400 nm; E, 800 nm.

**FIG. 3.**
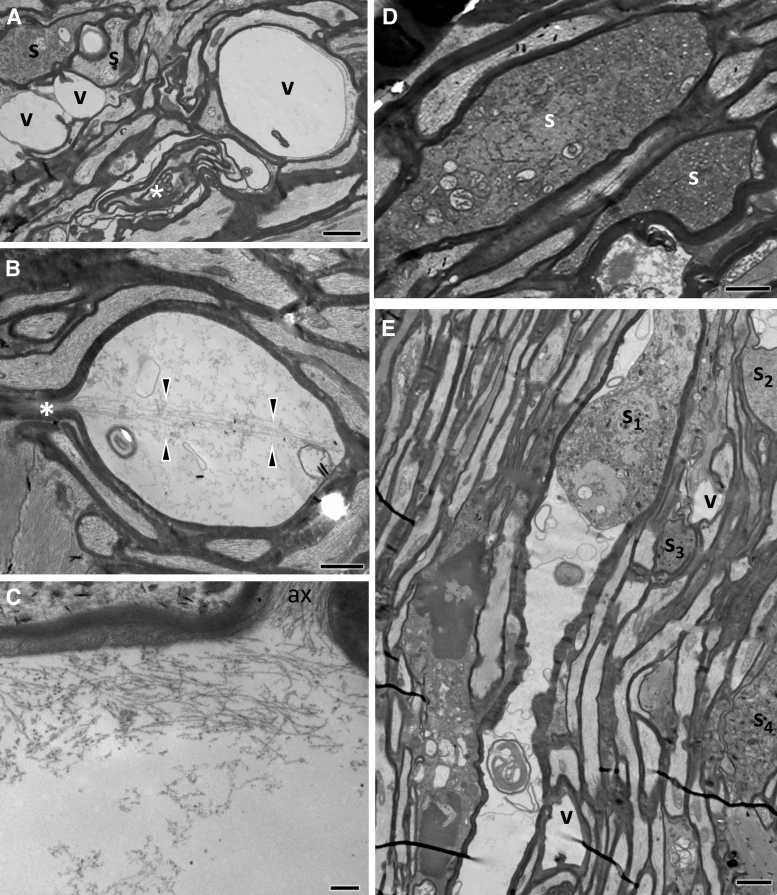
Ultrastructural features of primary traumatic axonal injury of the optic nerve (ON) 4 h after impact acceleration traumatic brain injury (IA-TBI). **(A)** There is severe vacuolar disruption (v) of the tissue and the formation of electrodense axonal swellings (s). There is grossly distorted axonal and myelin anatomy either because of degradation (*) or displacement by enlarged axonal segments. **(B)** A vacuolar axonal swelling containing floccular material in continuity with a better-preserved axon segment (*). There is partial preservation of neurofilaments projecting from the preserved axon segment (arrowheads). **(C)** A detail from another vacuolar axonal swelling in continuity with a better-preserved axon segment (ax) showing various degrees of axoskeletal digestion. Note the uniform alignment of neurofilaments within ax and the progressive disorganization and digestion in the vacuolar swelling. **(D)** Profiles of electrodense swellings (s) containing disorganized cytoskeletal elements and multilamellar and electrodense bodies. **(E)** Electrodense axon swelling (s_1_) contained within an otherwise preserved and hydropic axon bed with the appearance of an end bulb. Other electrodense (s_2-3_) and vacuolar swellings (v) are seen among normal appearing axons. Scale bars: (A) and (E), 2 μm; (B) and (D), 1 μm; (C), 200 nm.

In the early phase after injury (20 min and 4 h; Fig. 2 and Fig. 3, respectively), axonal and myelin sheath perturbations are restricted to the injury front, while the proximal and distal segments of the ON appear essentially normal. Mild pathological changes include profiles with accumulation of distended mitochondria and vesicles or vacuoles at the periphery of the axoplasm (Fig. 2A, 2B), focal cytoskeletal rarefaction, and some disruption of the myelin-axon interphase (Fig. 2B, 2D). There may also be free extrusion or axolemma-bound protrusion of nodal axoplasm (Fig. 2C, 2D) previously described as “nodal blebbing.”^39^ In more severely affected axons, there is gross disorganization of the axonal bed, including separation into hydropic areas that contain floccular material and membrane-bound segments of compacted axoplasm (Fig. 2E). At 4 h post-injury, the tissue architecture is grossly disrupted and some axons develop large vacuolar swellings (Fig. 3A-C). These swellings may contain cytoskeletal elements in various stages of digestion (Fig. 3B, 3C). At 4 h, besides profiles with compacted cytoskeleton there are also electrodense axonal swellings filled with vesicles, multilamellar and dense bodies, and normal-appearing or swollen mitochondria (Fig. 3D, 3E) These latter profiles correspond to previous descriptions of spheroids.^40^ End-bulbs of apparently disconnected axons at the proximal and distal retraction zones have identical ultrastructure (Fig. 3E).

**FIG. 4:**
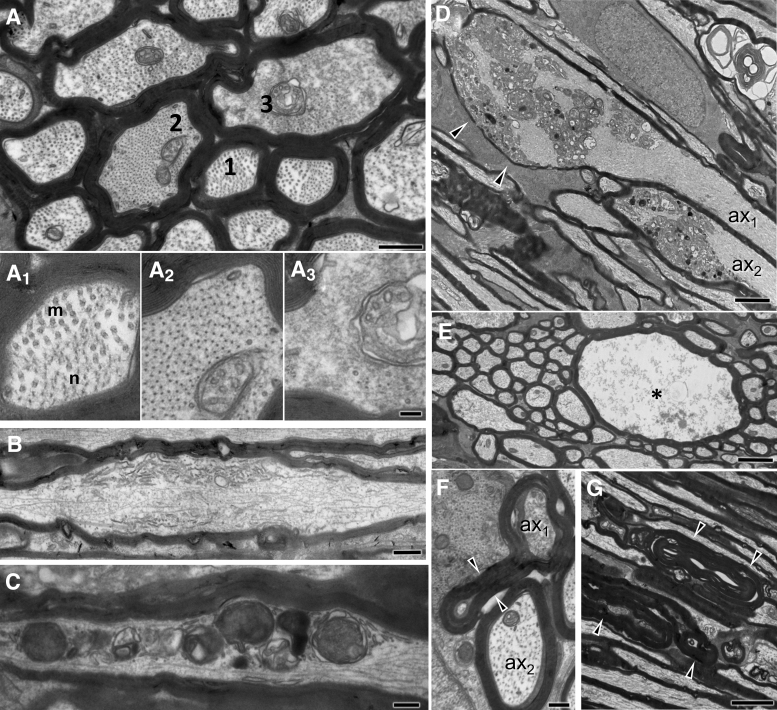
Ultrastructural features of optic nerve (ON) axonopathy 3 days following impact acceleration traumatic brain injury (IA-TBI). **(A)** Transverse section through the distal ON section showing normal axons (1) with preservation of microtubules (m) and neurofilaments (n) and a range of cytoskeletal changes including, neurofilament compaction and loss of microtubules (2), to cytoskeletal degradation (3). Note the distended mitochondria in profiles 2 and 3. Images at the bottom are details of profiles with the same numbers on top. **(B, C)** Axonal varicosities with focal sequestration of smooth endoplasmic reticulum (B) or dense bodies and other membrane-bound organelles (C). **(D)** Electrodense axonal swellings in continuity with better-preserved axon segments (ax_1-2_) in the proximal retraction zone. There is aggregation of organelles, dense bodies and compact neurofilament bundles in different orientations, with thinning of the ensheathing myelin (arrowheads). **(E)** Hydropic axon swelling with floccular content in the distal ON (*). **(F)** Excess myelin profiles (arrowheads) next to a degenerating and a normal-appearing axon (ax_1_ and ax_2_, respectively). **(G)** Longitudinal section showing myelin ovoid profiles (arrowheads) consistent with axon fragmentation. Scale bars: (A) and (B), 500 nm; (A1-3), 100 nm; (C) and (F), 250 nm; (D, E) and (G), 2 μm.

By 3 and 7 days post-injury, pathology has extended to distal and proximal axon segments. Pathological profiles are dense in the core of the ON, whereas the periphery is relatively spared. Gross tissue disruption related to vacuolar changes at the injury front is less apparent at these time-points (compare with Fig. 2A, 2B), but there are evident axonal and myelin changes consistent with progressive axonopathy (Fig. 4). Pathological profiles are often seen in contiguity with normal-appearing sections along the same axon. In less impacted axons, there is only circumscribed loss of microtubules and neurofilament compaction often surrounded by normal myelin sheaths (Fig. 4A) or accumulation/sequestration of organelles or other material (Fig. 4B, 4C).

Dense spheroids or apparent end bulbs comprised of greatly enlarged (up to 10 μm in diameter) axonal profiles with thin or absent myelin abound at the proximal and, to a lesser extent, the distal retraction zones. In the proximal ON, these late spheroids are characterized by a substantial accumulation of neurofilaments in dense bundles (Fig. 4D). In the distal ON there are also hydropic axonal swellings containing digested cytoskeletal elements (Fig. 4E). Myelin pathology includes the formation of excess myelin figures featured by normal-looking compact myelin in abnormal configurations, usually extending over some distance (Fig. 4F). In severe cases, there is collapse of axonal structure with the formation of ovoids, especially on Day 7 (Supplementary Fig. S1). Macrophage processes invariably colocalize with axonal or myelin pathology and some contain myelin remnants. On occasion, macrophage processes may invade the myelin sheaths of degenerated axons (Supplementary Fig. S1).

**FIG. 5.**
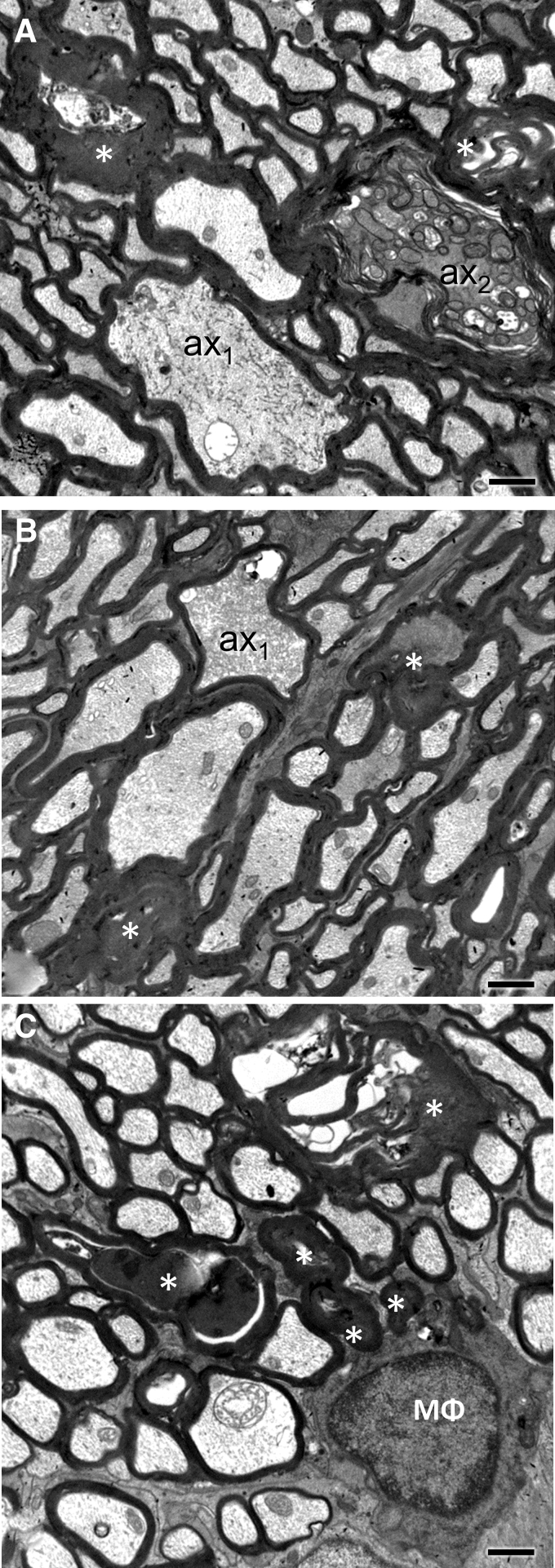
Ultrastructural features of traumatic axonopathy in optic nerves at 21 days after impact acceleration traumatic brain injury (IA-TBI). **(A-B)** Transverse sections through the distal optic nerve (ON) at 21 days show axons at different stages of degeneration. In (A) ax_1_ shows cytoskeletal compaction and aggregation of endoplasmic reticulum membranes while ax_2_ shows accumulation of degenerating mitochondria and other membranous organelles with an abnormally dense axoplasm and separation of myelin lamellae. In (B) ax_1_ is undergoing cytoskeletal dissolution. Ovoids (*) are common. **(C)** Myelin bodies and debris (*) colocalizing with a macrophage (ΜΦ). Scale bars (A-C), 1 μm.

By Day 21 post-injury, there are still axonal profiles at earlier stages of degeneration (Fig. 5A, 5B), while the bulk of pathology involves dark axons and, especially, collapsed or dense amorphous myelin profiles (Fig. 5B, 5C). An increasing number of end-stage pathological myelin profiles is colocalized with macrophage processes (Fig. 5C).

### Effect of *Sarm1* disruption on traumatic axonopathy in the optic nerve

In the previous two sections, we have demonstrated that IA-TBI causes acute perturbations of the axoskeleton, axolemma, and myelin sheath at the site of the initial biomechanical disruption, changes that evolve further over the course of days-weeks to a degenerative axonopathy distal and proximal to injury site, with morphological features reminiscent of models of WD and other neuropathies. Wallerian degeneration is present in diverse types of axonal injury from mitochondrial dysfunction to axonal transection and is executed via a SARM1-dependent program of axonal fragmentation, particularly in segments at the site of injury and distal to it.^23^ While there is mounting evidence for the protective effect of *Sarm1* interference on the degeneration of distal axon segments after axotomy,^41–43^ and in mixed populations of axons after TBI,^28,29,44-46^ the role of SARM1 on the degeneration of the proximal axon or retrograde neuronal death after TBI has not been established as of yet.

First, to better understand the course of degeneration and the role of SARM1 in traumatic axonopathy, we assessed the degree of apparent disconnection early after IA-TBI in the presence and absence of *Sarm1*. To this effect, we crossed *Sarm1* KO mice with Thy1-YFP-H mice and estimated the percentage of disconnected axons 24 h after IA-TBI in cleared ONs from littermate YFP-H and YFP-H/*Sarm1* KO mice. We found that IA-TBI led to axon disconnection in 37 ± 9 % and 47 ± 7 % of YFP labeled axons in each genotype respectively (*t_7.4_* = 2.17, *p* = 0.06), indicating that TAI-induced axon disconnection does not appear to depend on SARM1 activity.

**FIG. 6.**
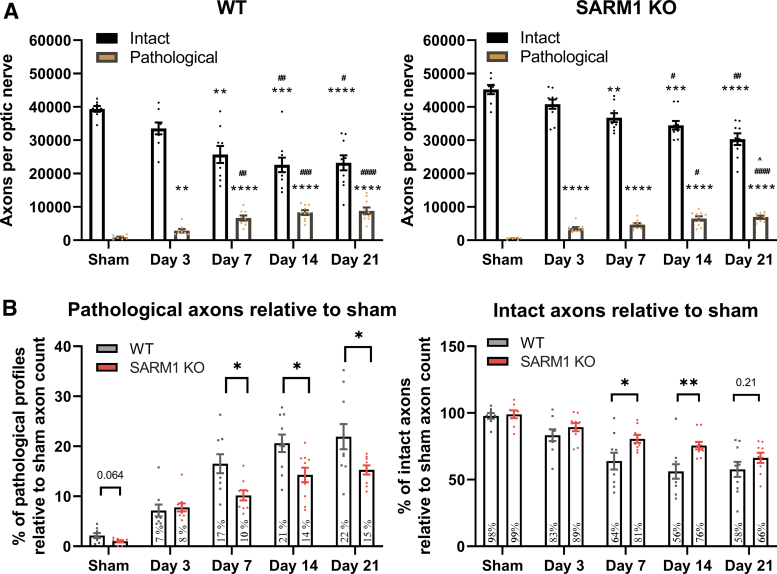
Assessment of intact and pathological axons in the distal optic nerves of wild-type and *Sarm1* knockout (KO) mice. Toluidine blue- stained semithin sections from distal optic nerve (ON) segments were assessed by stereology for the estimation of pathological and intact axon profiles (for criteria see the Methods section). **(A)** Impact acceleration traumatic brain injury leads to progressive degeneration of distal ON axons in both wild type and *Sarm1* KO mice. In wild type (wt) mice, there are reductions in intact axons compared with shams at 7 (*t_11.5_* = 2.98, *p =* 0.004), 14 (*t_9.7_* = 5.07, *p =* 0.001) and 21 days post-injury (*t_11.6_* = 7.14, *p =* 0.0003), while significant differences are also observed at 14 and 21 days relative to Day 3 (*t_16.6_* = 3.89 with *p =* 0.01 and *t_16.5_* = 3.60 with *p =* 0.022, respectively). Similarly, there are more pathological axon profiles relative to sham, at 3 (*t_10.6_* = 3.87, *p =* 0.023), 7 (*t_9.1_* = 7.34, *p =* 0.0004), 14 (*t_10._*_5_ = 10.1, *p* < 0.0001), and 21 days (*t_9.72_* = 7.68, *p =* 0.0002); and relative to Day 3, at 7 (*t_13._*_5_ = 4.19, *p =* 0.01), 14 (*t_15.5_* = 6.35, *p =* 0.001), and 21 days (*t_12.7_* = 5.29, *p =* 0.0014). In *Sarm1* KO mice, there is loss of intact axons compared with sham animals at 7 (*t_14.9_* = 4.39, *p =* 0.005), 14 (*t_17.9_* = 3.33, *p =* 0.0004) and 21 days (*t_14.6_* = 6.73, *p* < 0.0001); and relative to Day 3, at 14 (*t_17.9_* = 3.33, *p =* 0.035) and 21 days (*t_15.8_* = 4.69, *p =* 0.002). Pathological profiles also increased relative to sham animals at all time-points (*t* = 8.0-15, *p* < 0.0001), relative to Day 3 at 14 (*t_14_* = 3.9, *p =* 0.014) and 21 days (*t_16.4_* = 6.11, *p =* 0.0001); and relative to Day 7 at 21 days (*t_15.8_* = 3.67, *p =* 0.019). Relative to sham, **p < 0.01, ***p < 0.001, ****p < 0.0001; relative to Day 3, ^#^*p* < 0.05, ^##^*p* < 0.01, ^###^*p* < 0.001, ^####^*p* < 0.0001; relative to Day 7, ^*p* < 0.05. **(B)** *Sarm1* KO significantly delays intact axon loss and reduces the pathological burden. For direct comparisons between wt and *Sarm1* KO mice, intact and pathological counts are presented relative to total axon counts in sham animals of each genotype. *Sarm1* KO protects intact axons at both 7 and 14 days post-injury, but not at 21 days. Similarly, *Sarm1* KO reduces the pathological burden at 7, 14, and 21 days. See main text for details. *p < 0.05, **p < 0.01. Color image is available online.

Second, to test whether the evolving traumatic axonopathy in the ON is dependent on SARM1 we took advantage of the robust delineation of ON segments proximal and distal to the injury front and explored axonal pathology or viability at 3, 7, 14, and 21 days after injury on semithin preparations with stereological methods (Fig. 6). We found that, in wt mice, axonal pathology after IA-TBI reaches a plateau at 7 days and persists at that level until the end of the study. In addition, there is loss of ≈40% of intact axons that also reaches a plateau at 7 days (Fig. 6A). Although the ultrastructure of pathological profiles is similar between wt and *Sarm1* KO (SKO) mice (Supplementary Fig. S2), the course of degeneration in the latter is more protracted (Fig. 6A). Because *Sarm1* KO mice have more axons at baseline (Wt: 39398 ± 2424, SKO: 45211 ± 3856; *t_14_* = 3.61, *p* = 0.003), we standardized intact and pathological axon counts against the total number of axons from sham mice for each genotype (Fig. 6B). Given that SARM1-dependent degeneration may contribute differentially at various time-points or alter the course of degeneration, we assessed the effect of *Sarm1* KO by a two-way ANOVA for the effect of genotype, time and their interaction.

With respect to pathological profiles, we found that there is a significant effect of genotype (*F_1,82_* = 18.31, *p* < 0.0001), time (*F_4,82_* = 45.44, *p* < 0.0001), and their interaction, (*F_4,82_* = 2.78, *p* = 0.032), showing that *Sarm1* KO has significant effects on axonal pathology depending on time. Specifically, Holm-Šídák's multiple comparisons show significant differences at 7 (*t_82_* = 3.02, *p* = 0.01), 14 (*t_82_* = 3.20, *p* = 0.0079) and 21 days post-injury (*t_82_* = 3.27, *p* = 0.0079), indicating that *Sarm1* KO reduces pathology by 30-40 % at these time-points, but not at 3 days post-injury. With respect to intact axon counts, there is also significant effect of genotype (*F_4,82_* = 14.68, *p* = 0.0002) and time (*F_4,82_* = 23.96, *p* < 0.001) but not their interaction (*F_4,82_* = 1.52, *p* = 0.2). *Post hoc* multiple-comparisons testing shows significant differences at 7 (*t_82_* = 2.73, *p* = 0.03) and 14 days (*t_82_* = 3.34, *p* = 0.006) translating to about 50% protection, but not at 3 or 21 days post-injury. In the latter, it only shows a 20% trend protection (*t_82_* = 1.46, *p* = 0.38).

### Effect of *Sarm1* disruption on proximal axons and perikarya

**FIG. 7.**
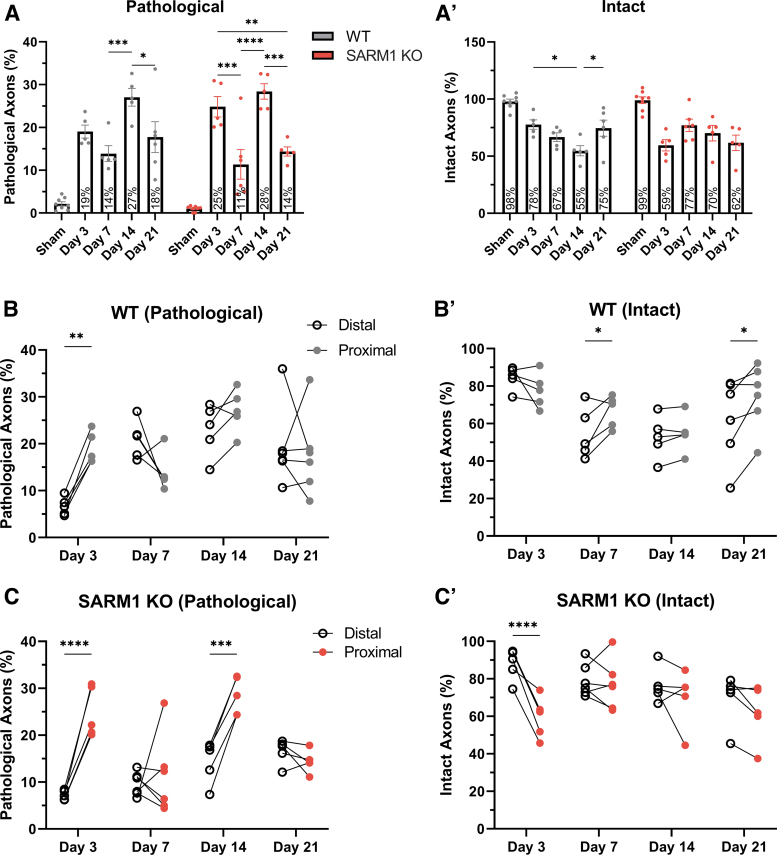
Differential effects of impact acceleration traumatic brain injury (IA-TBI) and *Sarm1* disruption on proximal and distal axonal pathologies. Analysis of pathological **(A, B, C)** and intact profiles (A′,B′,C′) was performed in both distal and proximal axon segments in wild type (wt) and *Sarm1* knockout (KO) mice. Mixed-effects analyses targeted on axonal pathology and axon loss show a significant effect of location (for pathology, *F_1,34_* = 20.8, *p* < 0.0001; for axon numbers, *F_1,34_* = 4.45, *p =* 0.042), and its interaction with genotype (*F_1,34_* = 7.46, *p =* 0.01; *F_1,34_* = 28.06, *p* < 0.0001) and time (*F_3,34_* = 16.06, *p* < 0.0001; *F_3,34_* = 3.47, *p =* 0.027). (A-A′) Here, pathological (A) and intact (A′) axon counts for proximal optic nerve (ON) are shown for each genotype. Two-way analysis of variance revealed no significant effect of genotype (Pathological: *F_1, 48_* = 0.0003, *p =* 0.99; Intact: *F_1, 48_* = 0.07, *p =* 0.8), but significant effect for time (*F_4, 48_* = 51.86, *p* < 0.0001; *F_4, 48_* = 20.92, *p* < 0.0001) and their interaction for intact axons only (*F_4, 48_* = 4.01, *p* = 0.007). *Post hoc* comparisons are shown graphically (for clarity, comparisons with sham are not shown, all *p* < 0.05). (B-B′) Counts between proximal (closed circles) and distal (open circles) segments from individual ON are shown for wt animals. Mixed effect analysis of pathological profiles (B) reveals significant effect of time (*F_3,1_*_7_ = 4.14, *p =* 0.02), location (*F_1,17_* = 6.06, *p =* 0.025), and their interaction (*F_3,17_* = 5.33, *p =* 0.009) due to greater axon losses distally at Days 7 (*t_17_* = 2.99, *p =* 0.025) and 21 (*t_1_*_7_ = 3.3, *p =* 0.017). Mixed effect analysis of pathological axons (B′) reveal significant effect of time (*F_3, 17_* = 6.29, *p =* 0.005) and its interaction with location (*F_3,17_* = 6.07, *p =* 0.005), but not location on its own, due to greater axon pathology proximally at Day 3 post-injury (*t_17_* = 3.65, *p =* 0.008). (C-C′) Counts between proximal (closed circles) and distal (open circles) segments from individual ON are shown for *Sarm1* KO animals. Mixed effect analysis of pathological profiles (C) reveals an effect for location, (*F_1,17_* = 33.17, *p* < 0.0001), time, (*F_3,17_* = 9.84, *p =* 0.0005), and their interaction (*F_3,17_* = 12.05, *p =* 0.0002) due to higher pathological burden proximally at Days 3 (*t_17_* = 6.36, *p* < 0.0001) and 14 post-injury (*t_17_* = 5.05, *p* < 0.0003). Mixed effect analysis of intact axons (C′) reveals a significant effect for location (*F_1,17_* = 23.62, *p =* 0.0001) and its interaction with time (*F_3,17_* = 6.42, *p =* 0.004), with multiple comparisons showing greater axon losses proximally at Day 3 post-injury (*t_17_* = 6.03, *p* < 0.0001). Color image is available online.

**FIG. 8.**
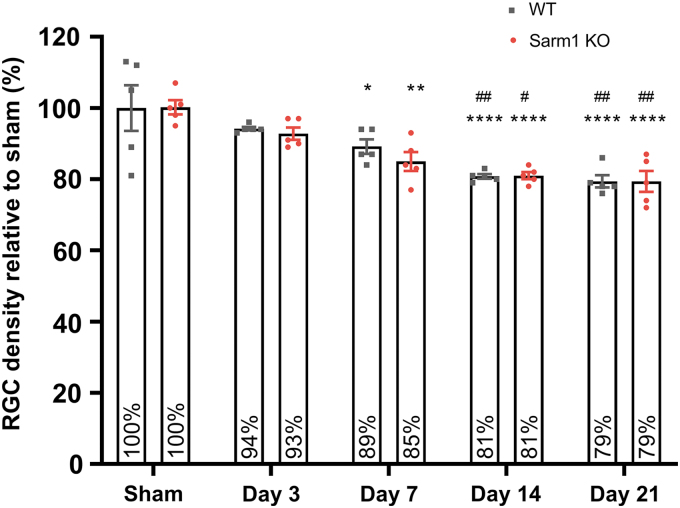
Loss of retinal ganglion cell somata after impact acceleration traumatic brain injury (IA-TBI) in wild type (wt) and *Sarm1* knockout (KO) mice. Densities of RGC somata were normalized to sham groups for each genotype. There is no difference in densities of RGC profiles between sham-injured wt and *Sarm1* KO retinas. Two-way analysis of variance with *post hoc* testing indicates a significant effect for time (*F_4,40_* = 20.80, *p* < 0.0001) but not genotype (*F_1,40_* = 0.37, *p =* 0.54). For both wt and *Sarm1* KO mice, there is loss of RGC somata relative to sham-injured mice at Day 7 (*p*_wt_ = 0.042; *p*_KO_ = 0.0022), 14 (*p* < 0.0001), and 21 (*p* < 0.0001). There is also a significant difference at Days 14 and 21 relative to Day 3 (*p*_wt_ = 0.007 vs. *p*_KO_ = 0.021 and *p*_wt_ = 0.003 vs. *p*_KO_ = 0.007, respectively). Relative to sham, *p < 0.05, **p < 0.01, ****p < 0.0001; relative to Day 3, ^#^*p* < 0.05, ^##^*p* < 0.01. Color image is available online.

After we established that *Sarm1* disruption leads to a significant protective effect on distal ON axons at 7, 14, and 21 days, we explored the role of SARM1 on proximal axons from the same ONs (Fig. 7) and also on RGC survival (Fig. 8) because of the known retrograde vulnerability of these nerve cells to experimental lesions.^22,47,48^

As in the case of ON portions distal to injury, IA-TBI causes pathological changes and loss of intact axons in the proximal ON in both wt and *Sarm1* KO mice at all time-points. However, in contrast to the ON segment distal to injury where there is progressive degeneration and loss of axons, in the proximal ON the numbers of intact and pathological axons show a surprising variance across time, that is, greater pathology and loss of intact axons on Days 3 and 14 than Days 7 and 21 (Fig 7A and 7A′). More importantly, a comparison of intact and pathological profiles between wt and *Sarm1* KO mice in the proximal ONs shows no effect of *Sarm1* KO on axonopathy. These observations suggest not only a distinct response of proximal axons to injury but also a lack of dependence on SARM1 activity.

We further confirmed the differential effect of *Sarm1* KO on the degeneration of ON segments proximal and distal to injury by comparing measures of neuropathy (pathology and axon loss) from the same ONs with mixed-effects analyses for the effect of location (distal versus proximal), genotype (wt versus *Sarm1* KO), time, and their interactions (Fig. 7). This analysis revealed a greater loss of axons distally compared with proximally at Days 7 and 21 in wt animals and less pathological burden in distal compared with proximal ONs of *Sarm1* KO mice at Days 3 and 14. Finally, assessment of proximal-to-distal ratios for pathological and intact axons further confirms the dynamic nature of the evolving axonopathy and the differential role of SARM1 in distal versus proximal axonopathy (Supplementary Fig. S3).

Because glutaraldehyde fixation poses challenges in immunohistochemistry for RGC markers, we estimated RGC densities from hematoxylin-stained sections based on established criteria of nuclear morphology for various retinal cell types (Fig. 8).^36,37^ We found that IA-TBI leads to progressive loss of RGC profiles in both genotypes, reaching a plateau by 14 days. For both wt and *Sarm1* KO mice, there is a 21% loss of RGC profiles by 21 days, with no differences between genotypes at any time-point (Fig. 8). Finally, comparisons of intact axon and RGC profile losses across time reveal a disproportionate degeneration of axons compared with somata, especially in wt animals (Supplementary Fig. S4).

### Sarm1 disruption ameliorates neuroinflammation associated with traumatic axonopathy

**FIG. 9.**
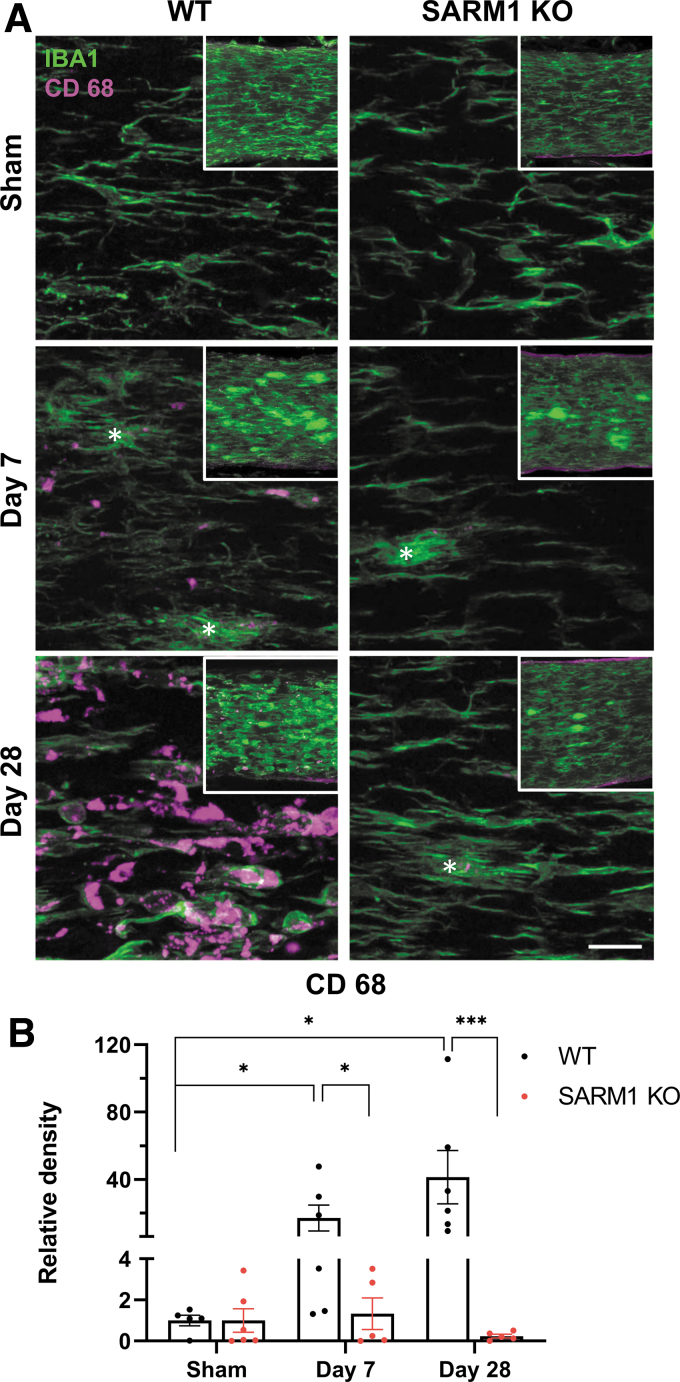
*Sarm1* disruption ameliorates microglial reactivity in the distal optic nerve after impact acceleration traumatic brain injury (IA-TBI). **(A)** Maximum projections of z-stack images (35 μm) showing IBA1 (green) and CD68 (magenta) immunoreactivity in the optic nerves of injured or sham-injured wild type (wt; left) and *Sarm1* knockout (KO) mice (right). Images were captured at 40 × , with insets (300 × 300 μm) showing maximum projections (60 μm) at lower power (20 × ). Activated microglia (white asterisks) in the ON appear to have a halo of IBA1- immunoreactivity as their processes interdigitate with intact and pathological axons (better resolved at the ultrastructural level as in Supplementary Fig. S1). Note the typically phagocytic amoeboid phenotype in wt ON at 28 days associated with strong CD 68 immunoreactivity. **(B)** Quantification of areal density of CD 68 immunoreactivity. Two-way analysis of variance reveals a significant effect of injury (*F_2,24_* = 3.63; *p =* 0.04), genotype (*F_1,24_* = 18.5, *p =* 0.0002), and their interaction (*F_2,24_* = 4.23, *p =* 0.027). *Post hoc* comparisons are shown *p < 0.05, *p < 0.001. Scale bar, 20 μm. Color image is available online.

Ultrastructural assessment indicated the presence of macrophages colocalizing with degenerating axons. Based on the finding that *Sarm1* disruption suppresses axon degeneration in the distal ON, we hypothesized that the reduced pathological burden would be associated with reduced neuroinflammation. To test this hypothesis, we assessed microglial activation and phagocytosis in the distal ON segment with immunohistochemistry for IBA1 and CD 68^49-51^ in cleared ONs. IBA1 immunoreactivity revealed profiles of activated microglia after injury in both genotypes (Fig. 9A). In wt mice, there was also a significant increase in CD 68 immunoreactivity at both early (7 days) and late time-points (28 days) after injury in wt, but not *Sarm1* KO mice (Fig. 9A, 9B), indicating that *Sarm1* disruption is associated with amelioration of the neuroinflammatory response. Interestingly, some CD 68(+) profiles in wt animals appeared to lack IBA1 immunoreactivity although more detailed examination of such profiles revealed very low expression of IBA1 (Supplementary Fig. S5). Ameboid IBA1(-), CD68(+) cells have been observed before in age-associated deep subcortical white matter lesions in humans, although their significance remains unknown.^52^

## Discussion

The findings of the present study support the idea that, in contrast to the all-or-nothing nature of transection injury (axotomy), TAI in the ON is featured by a variable involvement of individual axons ranging from apparent early disconnection of a subpopulation of axons to a range of ongoing axonal and myelin perturbations. The former are likely “passive” events from the direct destructive outcome of the biomechanical disruption whereas the latter represent an evolving “active” axonopathy resulting in the degeneration of a population of axons distal and proximal to the injury, along with retrograde death of a subpopulation of RGCs. The unique anatomical configuration of the visual system and a relatively clear delineation of the initial biomechanical trauma in the ON have enabled the characterization of the place of action of SARM1 in traumatic axonopathy in our model: SARM1 has a clear role in TAI-associated distal, but not in proximal axonopathy or in the retrograde degeneration of RGCs. *Sarm1* disruption reduces axonal degeneration in the distal optic nerve by up to 50% in the first 2 weeks after IA-TBI with a continued but lower effect at 3 weeks, but without evident effect on the initial TAI-related axonal disconnection. Axonal protection was also associated with reduction in microglial activation. These findings combined with morphological observations indicate that distal traumatic axonopathy in the ON has features consistent with WD.

Our study utilized unbiased sampling and stereological axon counts on toluidine-blue stained semithin sections that is the standard in the field.^35^ This methodology was chosen as the most appropriate because it can account for changes in packing density of axons and the non-uniformity of the injury. A caveat may be that it tends to favor larger myelinated axons and may underestimate smaller ones. This limitation may explain why *Sarm1* KO mice, which have slightly larger axon calibers, appear to have a higher number of axons compared with wt mice at baseline despite same number of RGCs (Supplementary Fig. S6). To address this, our counts of pathological and normal axons were controlled for these baseline differences. While a potential vulnerability of large-caliber axons after TBI has been suggested before,^33^ it would probably underestimate the effect of *Sarm1* KO but without affecting the conclusions of this study. Other variables such as injury severity, sex, and age, which may independently alter the vulnerability of axons to TAI and their responses to SARM1 manipulations, were outside the scope of the present study, but certainly deserve further investigation. Injury severity and the role of multiple hits were addressed in part in an earlier neuropathological study from our group based on Gallyas silver staining.^20^

Our morphological observations and axonal counts suggest that TAI of the ON is associated with two key features. First, the primary biomechanical insult and its immediate structural or physiological/biochemical consequences result in variable involvement of axons and in progressive degenerative changes that evolve over days and weeks. Second, different neuronal domains exhibit differential vulnerability, with distal axon segments degenerating more than proximal segments, and RGC somata.

With respect to the direct impact of IA, the disruption of tissue is rather pervasive but also variable for axons. It affects multiple cellular components including axons, oligodendrocytes (especially myelin sheath) and the BBB, but it does so to a different degree and with variable involvement of these constituents. Focal axoskeletal disruption and nodal blebbing and disruption of the axonal cytoskeleton or direct deformation of the myelin sheath have been previously reported in models of TAI *in vitro* and *in vivo*.^38,53–58^ Some of these changes have been attributed to focal mechanoporation of the axolemma, Ca^2+^ influx with subsequent mitochondrial overload and edema, and calpain-dependent degradation of cytoskeletal elements such as digestion of microtubules or loss of neurofilament side-arms leading to compaction.^59-62^ Acute increases in Ca^2+^ due to activation of calcium channels have also been implicated in the retraction of the proximal and distal axon segments in the first hours after injury termed acute axonal degeneration.^63-65^ Although we did not examine the three-dimensional ultrastructure of apparently separated axonal segments to confirm a true disconnection, discontinuities in Thy1-YFP labeled axons at 20 min and the apparent retraction of the proximal and distal axonal segments by 24 h in a subpopulation of axons (about 40% of YFP (+) axons in this particular transgenic) are consistent with this idea.

At later time-points (3 days to 21 days), morphological changes in the ON indicate an evolving axonopathy similar to what we have recently described in long tracts of the spinal cord^66^ and are also consistent with other models of TAI.^5,13,56,57,67^ As with acute TAI, traumatic axonopathy is also characterized by the variable involvement of the axon proper and the myelin sheath. Although we were not able to quantify the complexity and variety of the ultrastructural changes, observed profiles encompass a spectrum of pathological changes ranging from isolated axoskeletal or myelin sheath pathologies reminiscent of chronic neuropathies^68^ to typical WD profiles as described after ON transection or crush injury.^40,69,70^ It is noteworthy that while a majority of axons has degenerated by 7 days and at 21 days, and most pathological profiles are remnants of previously degenerated axons, there is still evidence of ongoing degeneration and active neuroinflammation. The latter may be, at least in part, due to the slow clearance of degenerated axons and myelin debris in the CNS,^71^ but it is also possible that partially injured, “metastable” axons^72^ initiate a WD program on an ongoing basis for weeks after the initial injury.^73^

Despite only minor morphological differences between the injured proximal and distal axon segments, axon counts disclosed some differences in responses across time. For instance, the number of intact axons in the proximal ON segment is significantly higher than in the distal segment, at least at 7 and 21 days, and more in register with numbers of surviving somata. Another challenge is a significant temporary increase in pathological proximal axon profiles at Days 3 and 14. Although we cannot exclude variability in the extent of the peri-traumatic area across different time-points, we hypothesize that these changes may relate to dynamic transitions between damage and recovery in the proximal axonal population, and the secondary degeneration of the proximal segment resulting from retrograde RGC death.^74^ On a related matter, we found that the attrition of RGC somata is less severe and more gradual than ON axons: at our study's end-point, only one RGC soma is lost for every two degenerated axons. The latter observation is in contrast to the nearly total loss of RGCs after proximal ON crush,^75,76^ and more in line with the partial loss of RGCs after pre-chiasmatic ON transection,^76^ indicating at least a role for the location of the lesion on the fate of RGCs if not for the different type of injury.

Since the discovery of SARM1 as the main trigger of axonal degeneration in classic models of WD,^42,77,78^ there has been a great drive to discern whether *Sarm1* deletion may also attenuate acute or chronic axonal pathology and degeneration in disease-related scenarios, including TAI.^73^ Our results confirm the central role of SARM1 in traumatic axonopathy and further clarify the cellular and temporal details of its involvement. The main finding of our study is that *Sarm1* disruption reduces both axon loss and pathology in the distal ON in the first 2 weeks after injury, with a continued effect on pathology at 3 weeks. The dependence of distal axon degeneration on SARM1 after TAI is generally consistent with findings from classical WD models such as ON crush, in which *Sarm1* KO or the closely related Wld^S^ mutation preserve the ability of transected axons to transmit action potentials for at least 5 days, and their structural integrity for up to 2 weeks.^42,79^

However, in contrast to axotomy, TAI also includes partially or segmentally injured axons that may survive in a SARM1-depedent metastable state, as recently shown in an *in vitro* model of rotenone toxicity.^80^ While we did not explore the effect of *Sarm1* KO beyond the time period of 3 weeks, Bradshaw and colleagues found that *Sarm1* KO mice had a higher number of intact and also fewer pathological axons compared with their wt counterparts in a mixed population of axons in the corpus callosum 10 weeks after TBI.^46^ Similarly, Maynard and colleagues reported that *Sarm1* KO mice also showed reductions in APP(+) lesions in some white matter tracts and attenuation of behavioral/cognitive deficits 6 months after repeated closed head injury.^44^ The fact that the protective effect on pathology at 3 weeks in our model is not accompanied by a significant effect on axon survival may be that this outcome is affected by the number of axons that passively succumb to acute disconnection within the first 24 h after injury that, as we showed, is not dependent on SARM1. We anticipate that milder traumatic impacts involving a smaller number of acutely disconnected axons and a corresponding increase in the number of metastable axons would reveal a clearer effect of *Sarm1* KO on survival even at later time-points. An important next step would be to define the population of metastable axons in our model with morphological or anatomical markers and perhaps restrict the exploration of protective effects of *Sarm1* KO on this key population.

Another key finding of our study is that *Sarm1* KO is selectively protective of distal, but not proximal, axons. There is also lack of protection of injured RGCs that is consistent with previous observations in *Sarm1* KO and *Wld^S^* mice after ON crush^42,79^ but in contrast to models of TNFα- or rotenone-induced RGC degeneration, in which *Sarm1* deletion is at least partially protective against cell loss.^81,82^ In our previous work, we have demonstrated that the degeneration of RGC somata and proximal, but not distal, axons depend on stress MAPK DLK/LZK signaling.^21,22,83^ Taken together, these findings suggest that the fate of the proximal and distal axonal segments after axonal injury depends on distinct molecular programs: the degeneration of the distal segment depends on SARM1 signaling, whereas the degeneration of the proximal segment and the RGC soma depend more on stress MAPK signaling (DLK-LZK).

While our overall results are consistent with prior work showing the effect of *Sarm1* KO on the fate of injured axons in models of TBI,^28,29,44-46^ there also are some notable differences. For example, it was previously shown that *Sarm1* KO significantly reduces APP(+) axonal profiles in the corpus callosum^28^ or bright fluorescent swellings in YFP-labelled axons in the brainstem^12^ acutely (2-48 h) after injury. In the current study, we found no effect of *Sarm1* KO on the rate of disconnected axons at 24 h or on axonal pathology and axon loss at 3 days in the optic nerve. However, markers of pathology are different between our current study and previous work. Specifically, terminal bulbs that may represent axon disconnections are not but a small subset of APP (+) pathologies or the more numerous varicosities (including spheroids) and undulations that arise on individual axon segments after injury. Expression of *Wld^s^* suppresses both acute axonal degeneration and the formation of spheroids after transection but does not prevent axonal retraction.^40,64^ Therefore, while *Sarm1* disruption may preserve the integrity of a transected axon, it may not influence the extent of early axon disconnection after TAI, or the ultimate fate of a disconnected axon.

Finally, *Sarm1* KO also mitigates injury-associated neuroinflammation. Traumatic axonopathy is associated with a strong neuroinflammatory phagocytic response which was observed at the ultrastructural level as well as with IBA1 and CD 68 immunohistochemistry. Microglial activation based on deramified IBA1(+) profiles was present in both genotypes after injury, but phagocytic CD 68(+) profiles that were abundant in injured wt mice were much less frequently encountered in *Sarm1* KO mice. This is at least in part secondary to the reduced axonal pathological burden in *Sarm1* KO mice.^82,84^ However, the magnitude of suppression of the CD 68 response to injury seems disproportionate to the observed reduction in axonal pathology by *Sarm1* KO. While this might be due to a non-linear relationship between axonal pathology and microglial activation, it also raises the question for a potential direct role of SARM1 in microglial activation, warranting further investigation.

## Conclusion

In summary, this is the first study to assess in parallel and with a comprehensive manner the effect of *Sarm1* KO on the fate of RGC axons proximal and distal to a traumatic impact and also its effects on RGC somata retrogradely affected by the injury. The relatively clear delineation of the biomechanical disruption front in the ON after injury with good demarcation of a proximal and distal axon segment and also the distant location of corresponding neuronal somata make it possible to demonstrate the presence of Wallerian mechanisms in distal axonal degeneration and nicely complement our previous molecular findings on the dependency of perikaryal and proximal axon degeneration on non-Wallerian (i.e., stress MAPK-related) processes.^22^ Together, this work may form the basis for novel approaches to treat traumatic axonopathy by separately targeting proximal and distal axonal domains.

## Transparency, Rigor, and Reproducibility Summary

This study was not pre-registered. Statistical power and sample size calculations were based on previous studies with *Sarm1* KO mice and an expected effect size at Day 7 after IA-TBI of 0.6 with regard to protection of intact axons. A sample size of nine subjects per group was planned and calculated to yield >90 % power to detect at least 50% protection in intact axons. Statistical power for the major secondary outcome measure of pathological profiles was >80%. Subjects were randomly assigned to groups and due to the scale of the project mice and tissues were processed in multiple batches. Experimental manipulations were performed with subjects in the fed state between 1 and 5 pm. Whenever possible care was taken to include multiple groups per batch. Analyses of experimental materials were performed by investigators blinded to relevant characteristics of the subjects. Wt and transgenic mice are available from Jackson Labs. While detailed in the method sections, further information on equipment and analytical reagents used to perform experimental manipulations are available upon request. The statistical tests used were based on the assumption of normality, and heteroskedeity in the case of CD68 quantifications was addressed by log-transformation of the data. The sample sizes and degrees of freedom reflect the number of independent measurements (i.e., mouse) while in the case of proximal-distal ON comparisons, non-independence of measurements has been addressed using mixed effect models as detailed in the methods section. Data are available upon request. A limited number of histological samples from each of the experimental groups are available for future analyses on request. The authors have agreed to publish the manuscript using the Mary Ann Liebert Inc. “Open Access” option under appropriate license.

## Supplementary Material

Supplemental data

Supplemental data

Supplemental data

Supplemental data

Supplemental data

Supplemental data
