# Supplementary material for: Traumatic Axonal Injury in the Optic Nerve: The Selective Role of SARM1 in the Evolution of Distal Axonopathy
Source: J Neurotrauma. 2023 Aug 16;40(15-16):1743–61. doi: 10.1089/neu.2022.0416 (PMC10460965; doi:10.1089/neu.2022.0416)

**Supplementary figure 1.**  Presence of macrophages and degradation of axon and myelin debri in the optic nerve of wild type animals after IA-TBI. **A-B.** Transverse sections through the core of an ON 7 days after IA-TBI showing abundance of pathological axon profiles, collapsed myelin figures and ovoids, and their co-localization with macrophages (ΜΦ) and their processes. Macrophage processes seem to occupy most of the free space between axons. Note the radial distortion of axons in (B). **C-D′.** There are ovoids (ov) and axons at different stages of degeneration (yellow overlay) and active phagocytosis and degradation. Note the extensive rough endoplasmic reticulum (arrowheads) that is typical of macrophage cytoplasm (blue overlay) and the formation of highly electrodense bodies within them (*) which may arise from myelin digestion. In (D-D′) note the unambiguous invasion of a myelin tube that still appears compact (red overlay).

Scale bars, all 2 μm.


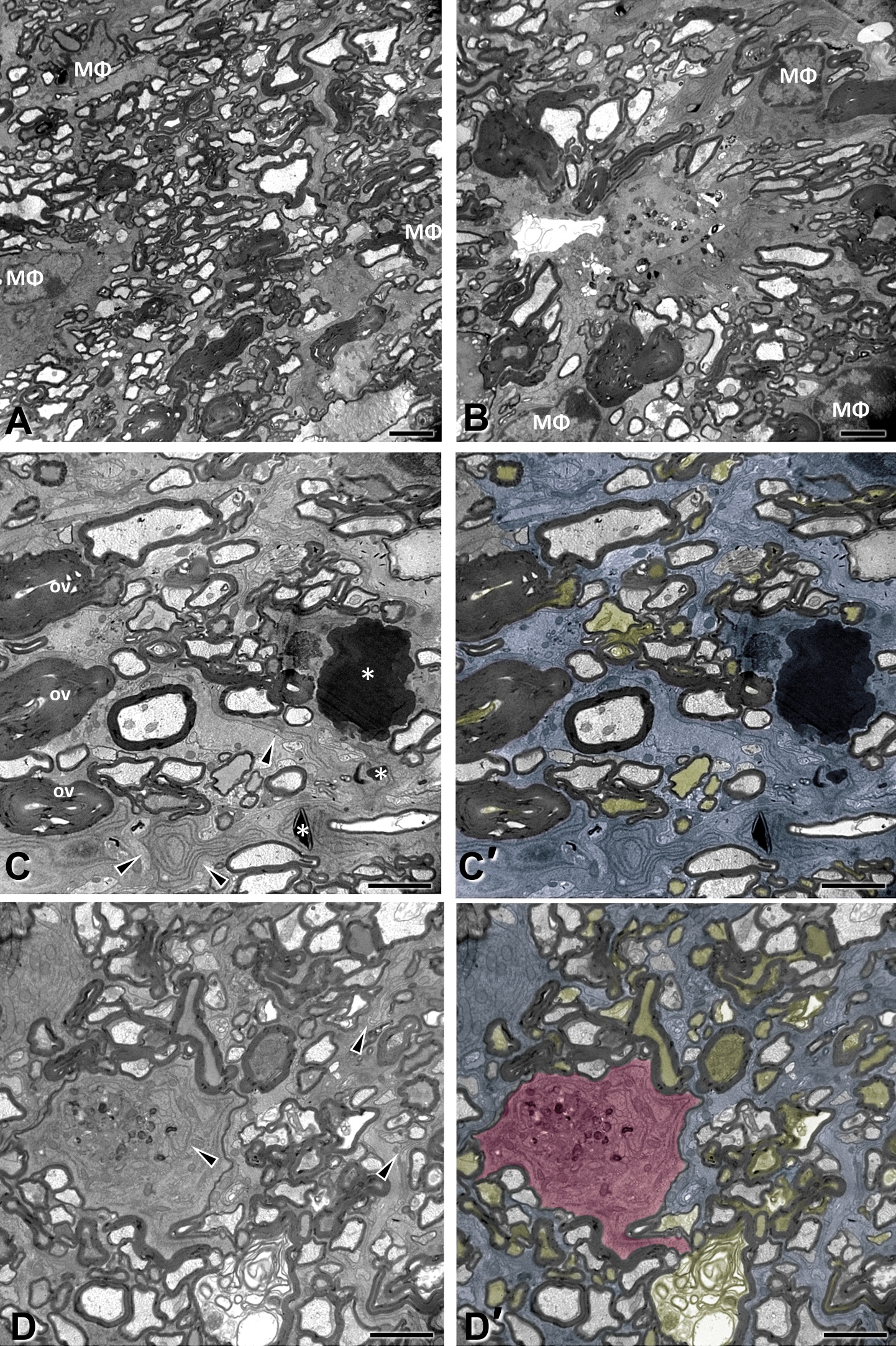

Supplement: Supplemental data [file Supp_FigS1.docx]
