# Supplementary material for: Traumatic Axonal Injury in the Optic Nerve: The Selective Role of SARM1 in the Evolution of Distal Axonopathy
Source: J Neurotrauma. 2023 Aug 16;40(15-16):1743–61. doi: 10.1089/neu.2022.0416 (PMC10460965; doi:10.1089/neu.2022.0416)

**Supplementary figure 2.** Ultrastructural features of axonal pathology in the optic nerve of *Sarm1* KO mice after IA-TBI. Transverse sections through the distal ON at 7 (A-C) and 21 days (D-F) after IA-TBI. In A and D, pathological axon profiles (*) show a range of features similar to those present in wt mice (See Fig. 4, Fig. 5 and Supplementary Fig. 1) ranging from cytoskeletal compaction and degradation to vacuolar swellings (v) and electrodense spheroids (s). Degenerating myelin is also seen (arrowhead). In B and E, macrophage processes (ΜΦ and *) are abundant and intermingle with intact and degenerating axons. Large spheroids (s) are typical at day 7 (C), while collapsed myelin sheaths and ovoids (white asterisks) are typical at day 21 (F). Scale bars, all 1 μm.


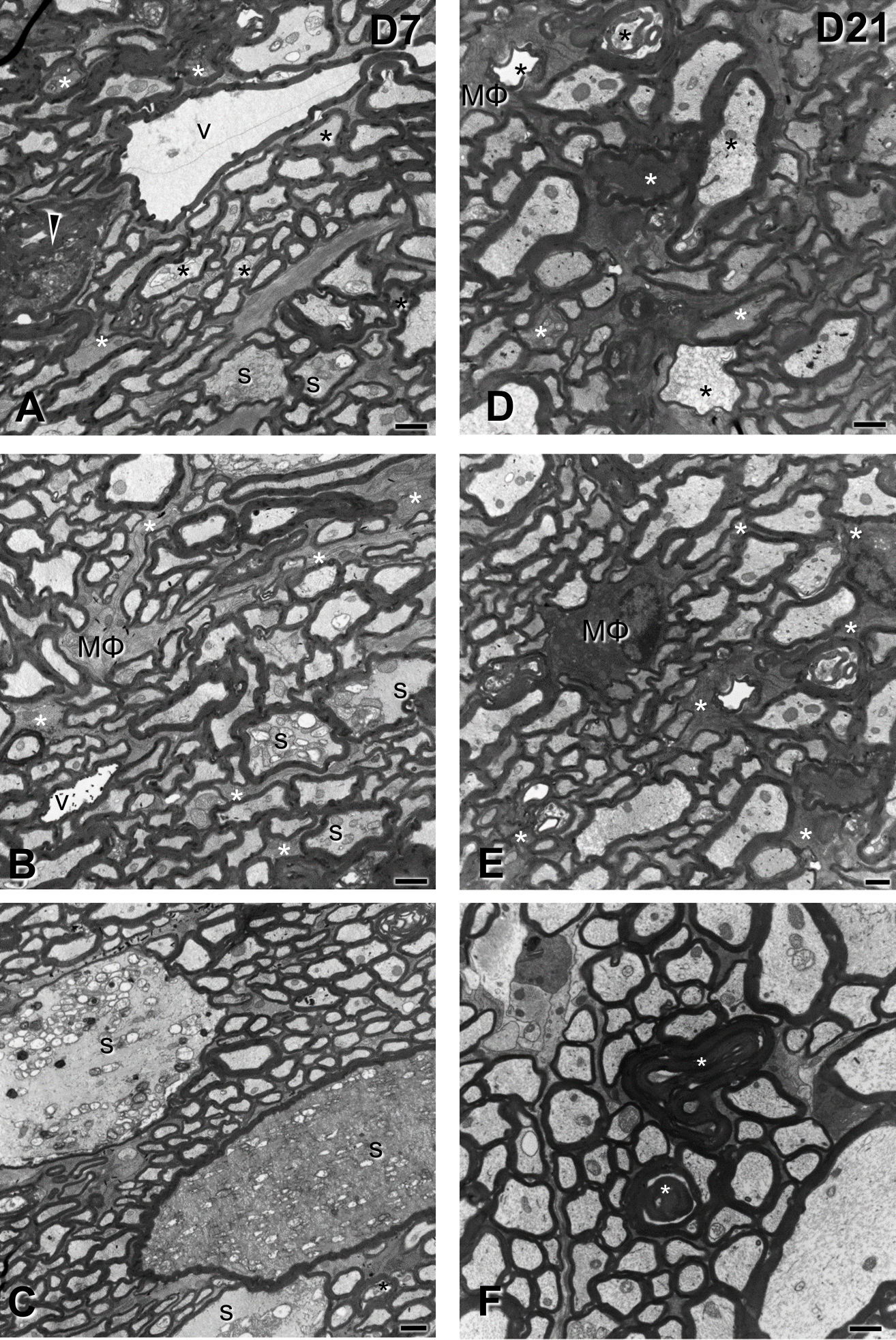

Supplement: Supplemental data [file Supp_FigS2.docx]
