# Supplementary material for: Traumatic Axonal Injury in the Optic Nerve: The Selective Role of SARM1 in the Evolution of Distal Axonopathy
Source: J Neurotrauma. 2023 Aug 16;40(15-16):1743–61. doi: 10.1089/neu.2022.0416 (PMC10460965; doi:10.1089/neu.2022.0416)

**Supplemental Figure 3.** Proximal-to-distal ratios of pathological (A) and intact (B) axons per ON for wt and *Sarm1* KO mice. Error bars represent 95% confidence intervals. Genotype has a significant effect on proximal/distal ratios for intact axons, *F_1,33_*= 22.01, *p*< 0.0001, but not pathological profiles *F_1, 33_*= 4.00, *p*=0.054. Statistical comparisons for each time point are also shown (unadjusted). * *p*<.05, *** *p*<.001.


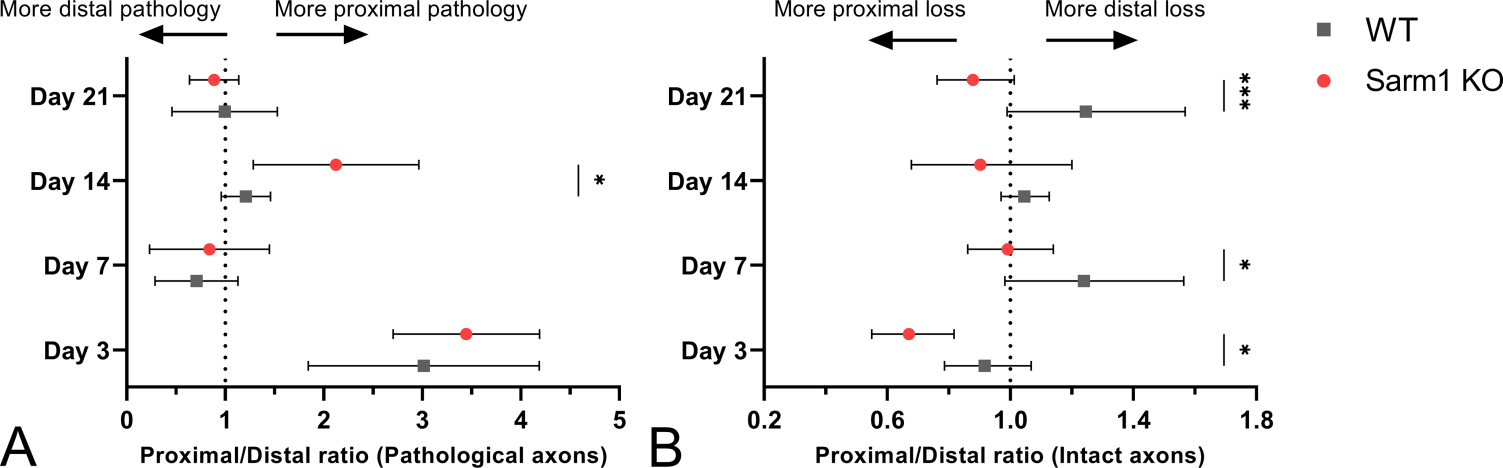

Supplement: Supplemental data [file Supp_FigS3.docx]
