# Supplementary material for: Traumatic Axonal Injury in the Optic Nerve: The Selective Role of SARM1 in the Evolution of Distal Axonopathy
Source: J Neurotrauma. 2023 Aug 16;40(15-16):1743–61. doi: 10.1089/neu.2022.0416 (PMC10460965; doi:10.1089/neu.2022.0416)

**Supplementary Fig. 4**. Comparison of cell body and distal and proximal intact axon attrition across time in wt and *Sarm1* KO mice. In wt but not *Sarm1* KO mice distal axon loss is significantly higher than cell body loss at 7, 14 and 21 days (* *p*<.05, *** *p*<.001). Significant differences between proximal axon and cell body attrition are also indicated (^#^ *p*<.05, ^##^ *p*<.01, ^####^ *p*<.0001).


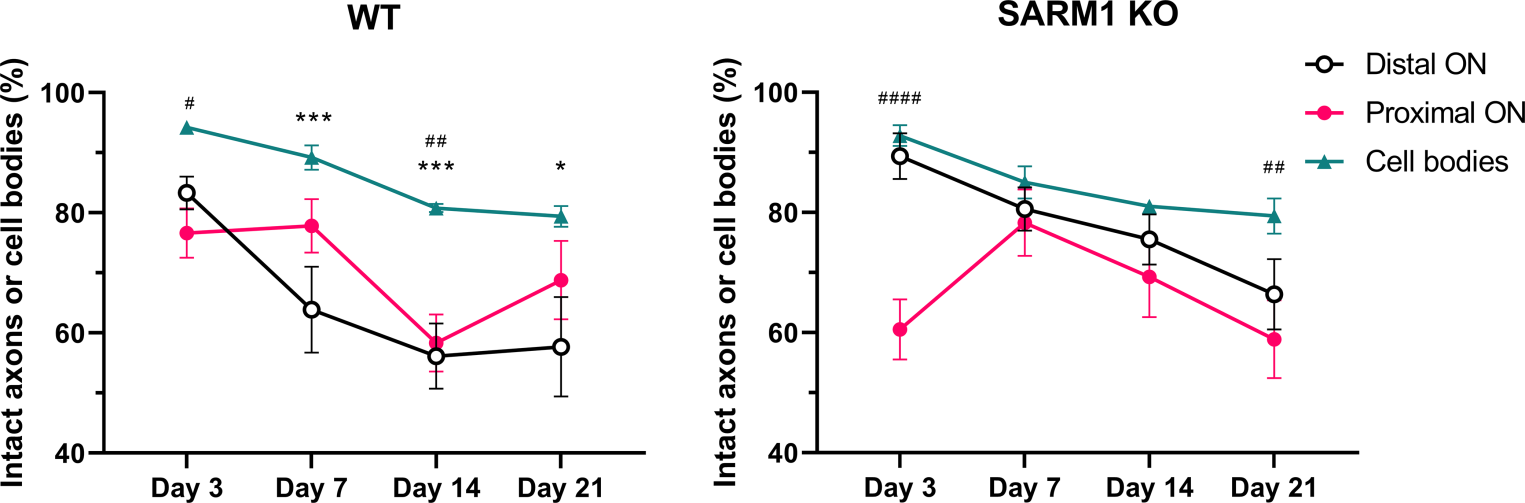

Supplement: Supplemental data [file Supp_FigS4.docx]
