# Supplementary material for: Traumatic Axonal Injury in the Optic Nerve: The Selective Role of SARM1 in the Evolution of Distal Axonopathy
Source: J Neurotrauma. 2023 Aug 16;40(15-16):1743–61. doi: 10.1089/neu.2022.0416 (PMC10460965; doi:10.1089/neu.2022.0416)

**Supplementary Fig.5.** IBA1 expression in CD68+ cells after IA-TBI. As a subpopulation of CD68+ cells in injured wildtype optic nerves (see Figure 9 and related text) appear to lack IBA1 immunoreactivity, we assessed line intensity plots from such profiles. A. Optical section showing IBA1 and CD68 immunoreactivity in a wt ON 30 days after TBI; white box (B) shows a representative CD68+ profile with low IBA1 expression. Monochromatic images of each channel corresponding to the same area are shown in panel (B), while the line intensity plot of the CD68+ profile (dotted line) is shown in panel (C).


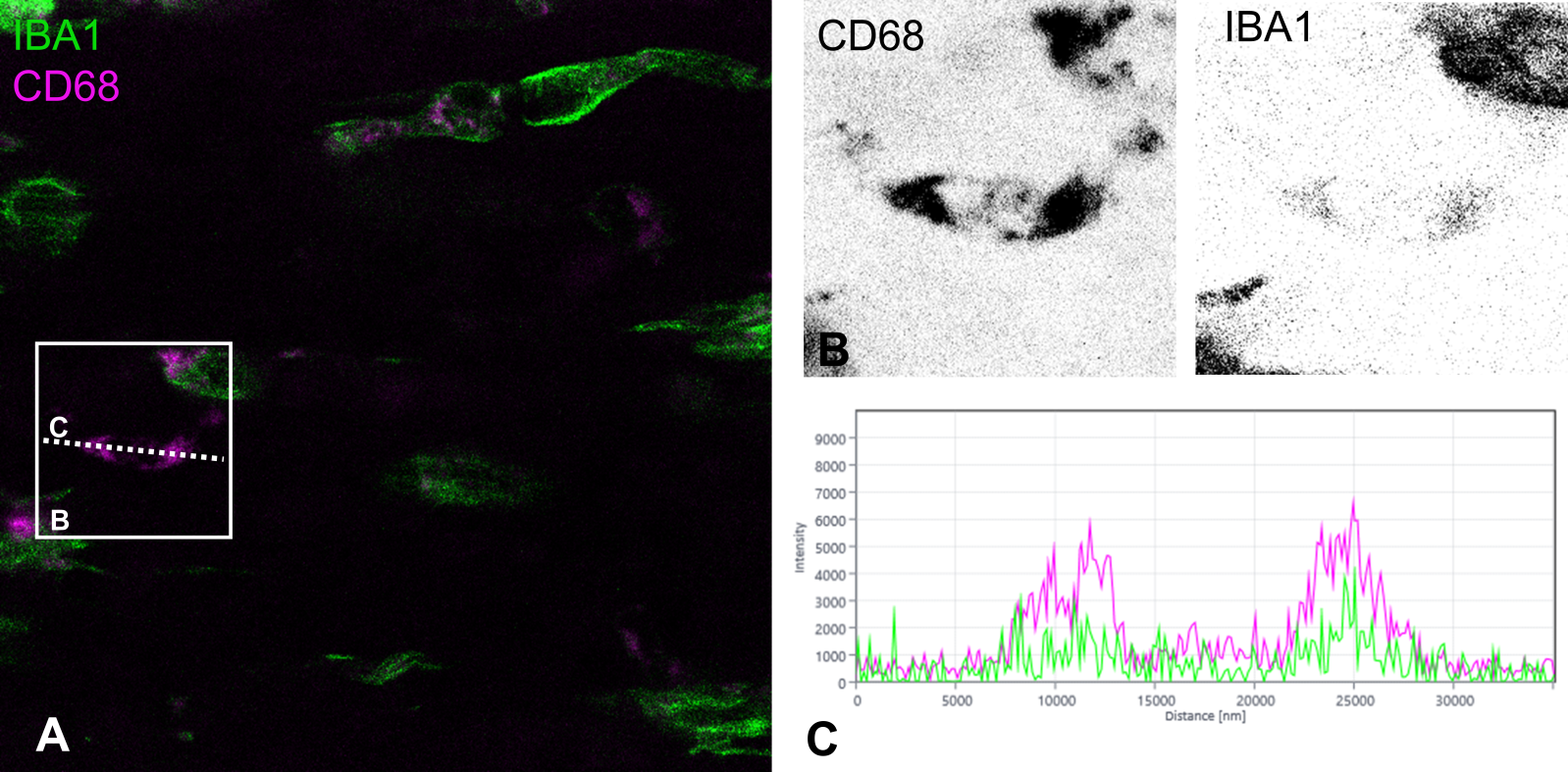

Supplement: Supplemental data [file Supp_FigS5.docx]
