# Supplementary material for: Traumatic Axonal Injury in the Optic Nerve: The Selective Role of SARM1 in the Evolution of Distal Axonopathy
Source: J Neurotrauma. 2023 Aug 16;40(15-16):1743–61. doi: 10.1089/neu.2022.0416 (PMC10460965; doi:10.1089/neu.2022.0416)

**Supplementary Fig. 6**. Axon diameter distribution analysis of sham-injured wt and *Sarm1* KO mice. Axon calibers were extracted from electron micrographs as per a previous paper from our group^31^ in order to plot relative frequencies. *Sarm1* KO animals tend to have higher frequencies of large caliber axons compared to wt animals (see also Figs. 1M and 3E by Marion et al. ^13^) but the same number of RGCs (see Fig. 8). The baseline difference in axon calibers explains the higher axon counts in KO animals when estimated with optical microscopy and the need to standardize to sham for each genotype when comparing wt with *Sarm1* KO animals (dotted lines represent potential detection threshold).


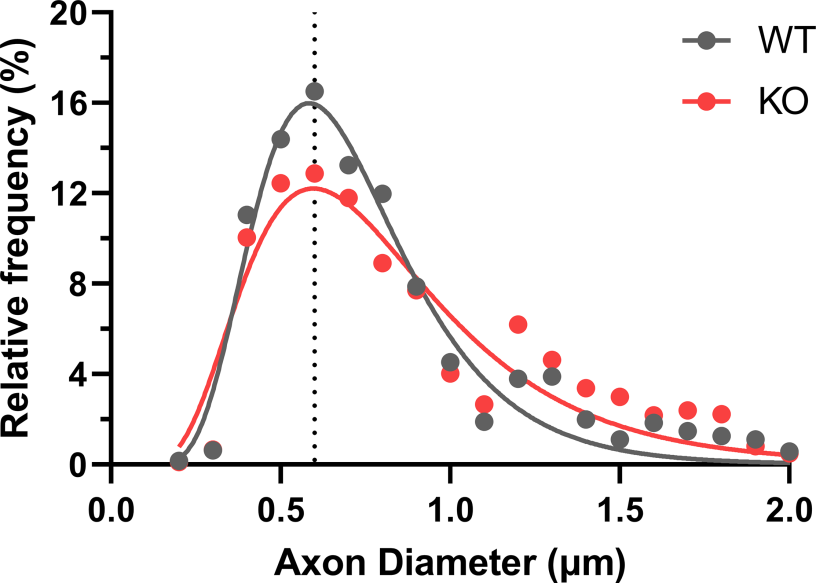

Supplement: Supplemental data [file Supp_FigS6.docx]
